# Systemic Lupus Erythematosus Associated with Erythema Multiforme-Like Lesions

**DOI:** 10.1155/2013/212145

**Published:** 2013-04-18

**Authors:** Ralph Yachoui, Patrick M. Cronin

**Affiliations:** Division of Rheumatology, Cooper Medical School of Rowan University, Voorhees, NJ 08043, USA

## Abstract

Erythema multiforme (EM) and systemic lupus erythematosus (SLE) are common diseases. Their coexistence is known as Rowell syndrome (RS), first described in 1963. Only few cases of RS have been described and some of them questioned its existence. We present two cases of SLE in the setting of a newly developed EM-like eruption, which shares many similarities with the so-called Rowell syndrome.

## 1. Introduction

 Systemic lupus erythematosus (SLE) has been rarely associated with erythema multiforme- (EM-) like eruption. In 1963, Rowell et al. described a distinctive subset of patients diagnosed with discoid lupus erythematosus (DLE) associated with EM and a characteristic pattern of immunological abnormalities (Table 1) [1]. We report two cases of SLE in the setting of a newly developed EM-like eruption, which shares many similarities with the so-called Rowell syndrome (RS).

**Case 1**

**Figure 1:**
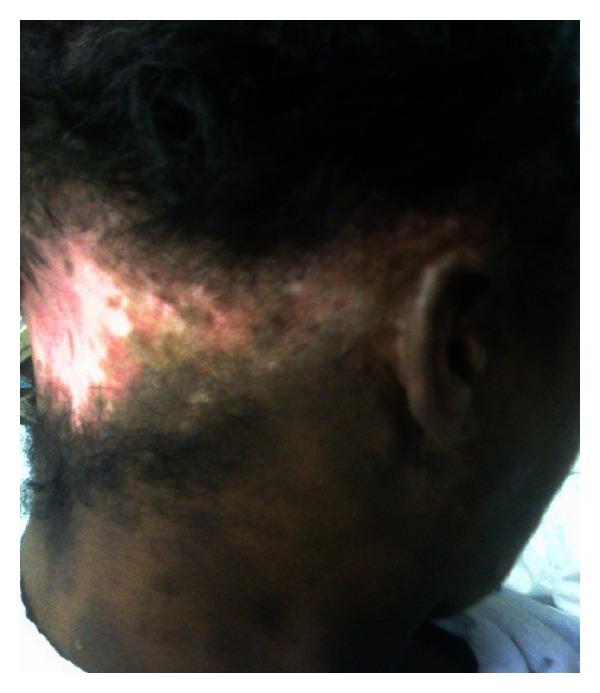


**Figure 2:**
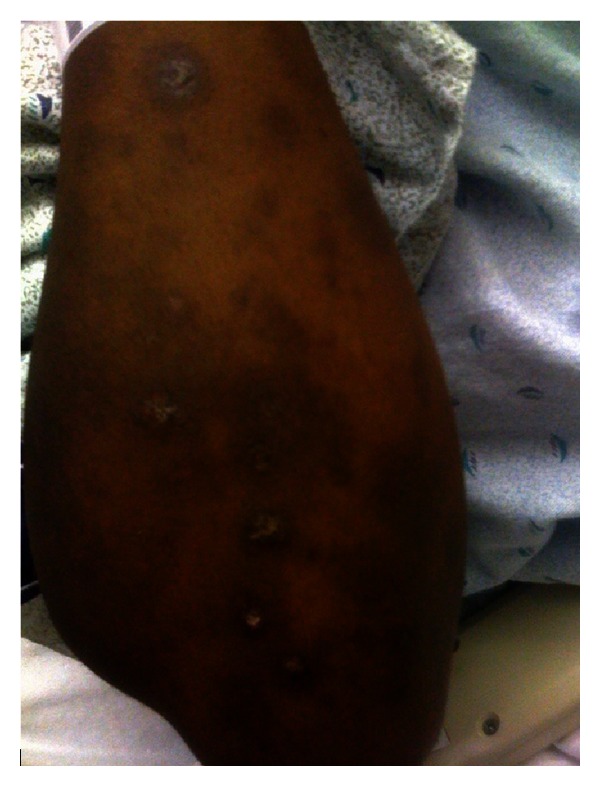


A 29-year-old African-American woman, with history of stroke and seizure disorder, presented with a 2-week history of diffuse rash without systemic symptoms and no apparent precipitating event. She had been taking phenytoin 100 mg three times daily for 2 years. Physical examination revealed scarring hypopigmented alopecia with follicular plugging and adherent scale extending from the ear lobes to the occipital scalp, clinically consistent with discoid lupus erythematosus (DLE) (Figure 1). There were also scattered erythematous to violaceous papules and plaques, mostly annular, some with scales, which coalesced into large polycyclic lesions, present on the chest, abdomen, arms, and legs with an overall appearance of subacute cutaneous lupus erythematosus (SCLE) (Figure 2). There were no oral, genital, or ocular lesions. The clinical impression indicated the coexistence of DLE and SCLE. However, a subsequent 4 mm punch biopsy of the SCLE-like lesions revealed histological hallmarks of erythema multiforme (EM), including perivascular lymphocytic infiltrate and widespread keratinocyte necrosis. Further workup demonstrated a 1 : 640 antinuclear antibody (ANA) with speckled pattern, anti-SSA > 8 (normal < 1), anti-SSB > 8 (normal < 1), C3 27 (normal 86–184 mg/dL), and C4 6 (normal 20–59 mg/dL). Rheumatoid factor was negative. A course of methylprednisolone at a dose of 120 mg IV daily was initiated. Hydroxychloroquine 400 mg daily was added. The lesions started to resolve 3 days later and the dose of methylprednisolone was gradually tapered within a period of 5 weeks.

**Case 2**

**Figure 3:**
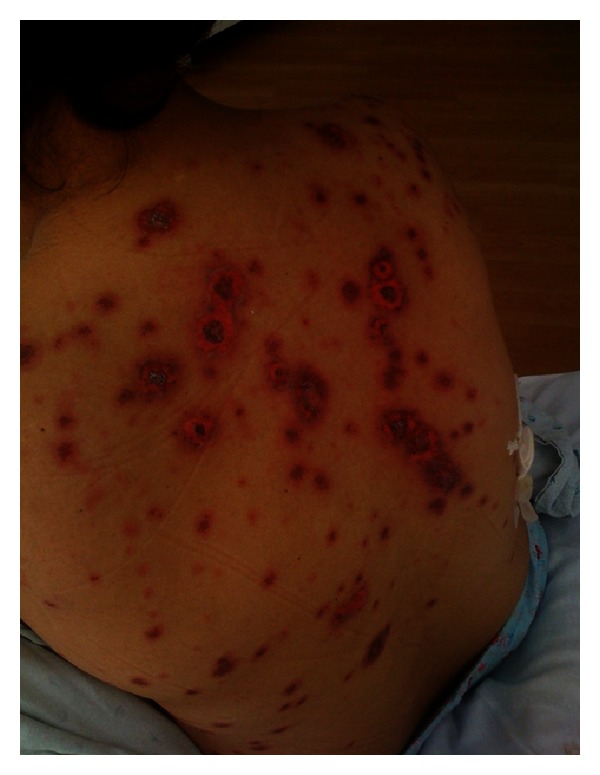


A 46-year-old Hispanic woman presented with 1-month history of widespread skin rash and fever. Lesions had first started on the arms, subsequently spreading to the upper anterior chest, back, and limbs. There was no apparent precipitating event. Physical examination revealed numerous erythematous, annular, and scaly plaques that coalesced on the anterior chest, back, and upper extremities (Figure 3). Her clinical picture was more consistent with SCLE. A biopsy of the SCLE-like lesions revealed areas of invasion with mononuclear cells and epidermis cell necrosis, findings consistent with EM. Complete blood count (CBC) demonstrated low white blood cell count at 1700/uL (normal 4000–11000/uL) with low absolute lymphocyte count at 450 (normal > 1500), a hemoglobin level of 8.5 g/dL, and a platelet count of 157 × 10^6^/L. The direct Coombs test was positive suggestive of autoimmune hemolytic anemia (AIHA). Additional work-up revealed a 1 : 640 antinuclear antibody (ANA) with speckled pattern, anti-Smith > 8 (normal < 1). Rheumatoid factor, anti-SSB, and anti-SSA antibodies were all negative. The patient was subsequently treated with methylprednisolone at 60 mg IV daily for 5 days, changed to prednisone 40 mg PO daily. The rash along with the pancytopenia started to resolve 1 week later.

## 2. Discussion

RS has first been described by Rowell in 1963 [1], who reported four cases of a syndrome characterized by SLE with EM-like lesions, speckled antinuclear antibodies, anti-SjT antibodies, and rheumatoid factor. Anti-SjT antibody is now thought to be identical with anti-La (SSB) antibody [2]. Since this original description, RS has been proposed in over 37 patients, although not always respecting the initial criteria [3–6]. Zeitouni and coworkers reviewed the diagnostic criteria for RS [6]. They proposed three major and three minor criteria. The major criteria consist of the presence of lupus erythematosus (systemic, discoid, or subacute lupus), EM-like lesions (with or without involvement of mucous membranes), and speckled pattern of antinuclear antibody. The minor criteria include chilblains, anti-Ro and/or anti-La antibodies, and positive RF. All three major and at least one minor criteria are required to establish the diagnosis of RS. 

EM is thought to fall within a spectrum of diseases that affect the skin and mucous membranes, including erythema multiforme, Stevens-Johnson syndrome, and toxic epidermal necrolysis [7]. Erythema multiforme is usually precipitated by infections (e.g., herpes simplex and mycoplasma) and drugs (e.g., penicillin and sulfamide) and is generally not associated with any specific autoimmune abnormality. The skin lesions are often quite targetoid [7]. Therapy varies from simple observation to acyclovir and steroids on an empirical basis [7].

SCLE is an entity, which was first described by Sontheimer et al. in 1979 [8], as a distinct subset of cutaneous lupus that is characterized by psoriasiform and/or annular lesions in sun-exposed areas, absent or mild systemic involvement, and presence of circulating anti-Ro (SSA) antibodies and is frequently associated with the presence of human lymphocyte antigen (HLA)-DR3 [9]. SCLE has a peak age of onset in the fourth decade. The SCLE skin lesions may be associated with malar eruptions and discoid lesions [10].

Both our patients had SLE in association with a speckled pattern of antinuclear antibodies and histological evidence of erythema multiforme favoring the diagnosis of RS according to previous reports [3–6]. However, the clinical impression in both our patients was more consistent with lesions of SCLE. 

In fact, the clinical and histological differentiation of SCLE and EM may be difficult. Early lesions of annular-polycyclic pattern of SCLE may resemble EM. On the other hand, necrotic keratinocytes frequently seen in EM lesions may also be found in SCLE lesions [11]. Herrero at al. [12] found necrotic keratinocytes histologically in 6 of 13 (46%) SCLE patients. Such overlap between SCLE and EM has been reported by Mendonca [13], in which repeated biopsies of lesions previously read as EM showed SCLE instead.

From this, it can be concluded that the clinical, histological, and immunological findings overlap in RS and SCLE. We postulate that lupus erythematosus with EM-like rashes designated as RS represent a subset of SCLE with targetoid lesions, rather than a distinct entity.

## Figures and Tables

**Table 1:** 

Original criteria for Rowell syndrome
(1) Lupus erythematosus
(2) Erythema multiforme
(3) Immunological abnormalities in the serum
Speckled pattern of antinuclear antibody (ANA)
Anti-LA (SSB) antibody
Positive rheumatoid factor (RF)
